# Bioinformatics process management: information flow via a computational journal

**DOI:** 10.1186/1751-0473-2-9

**Published:** 2007-12-03

**Authors:** Lance Feagan, Justin Rohrer, Alexander Garrett, Heather Amthauer, Ed Komp, David Johnson, Adam Hock, Terry Clark, Gerald Lushington, Gary Minden, Victor Frost

**Affiliations:** 1 Information and Telecommunication Technology Center, University of Kansas, Lawrence, Kansas, USA

## Abstract

This paper presents the Bioinformatics Computational Journal (BCJ), a framework for conducting and managing computational experiments in bioinformatics and computational biology. These experiments often involve series of computations, data searches, filters, and annotations which can benefit from a structured environment. Systems to manage computational experiments exist, ranging from libraries with standard data models to elaborate schemes to chain together input and output between applications. Yet, although such frameworks are available, their use is not widespread–*ad hoc *scripts are often required to bind applications together. The BCJ explores another solution to this problem through a computer based environment suitable for on-site use, which builds on the traditional laboratory notebook paradigm. It provides an intuitive, extensible paradigm designed for expressive composition of applications. Extensive features facilitate sharing data, computational methods, and entire experiments. By focusing on the bioinformatics and computational biology domain, the scope of the computational framework was narrowed, permitting us to implement a capable set of features for this domain. This report discusses the features determined critical by our system and other projects, along with design issues. We illustrate the use of our implementation of the BCJ on two domain-specific examples.

## Introduction

The Bioinformatics Computational Journal (BCJ) is an extensible environment that integrates computational resources, methods, and data. Bioinformatics and computational biology span a wide variety of applications ranging from interpretation of gene expression data to protein structure prediction (For brevity, in this report we often describe *bioinformatics and computational biology *as bioinformatics.). However, within this range of applications there is considerable common ground that is pivotal to a domain-oriented approach. Many bioinformatics applications are centered on the rapidly growing sequence data archived at national centers [1]. These data can be seen as enablers of bioinformatics approaches. As a result, standard approaches to process these data have appeared, but specifics of the applications can vary widely. Foremost in common is sequence alignment [2]. Many other applications are widely used (for example, see EMBOSS [3]) and more are under development.

The BCJ is designed around characteristics of bioinformatics research, which often involves: (1) stepwise application of a series of methods, (2) re-application of the series of methods in search and retry iterations using different parameters, (3) development of new procedures and combinations in the rapidly growing discipline, (4) an open environment crucially dependent on web-based application servers and public databases, and (5) rapidly growing data collections and evolving vocabularies. The volume of data and the number of computational experiments create significant processing and management complications.

The BCJ framework reported here addresses these problems through data and process management, with mechanisms for experiment specification and composing computations, tracking data provenance and retaining the context of computations. Activities such as entering and locating data, applications, and results are facilitated by the framework. The BCJ fosters collaboration among project teams through structured privacy mechanisms and naming constructs, increasing productivity in computational experiments for both computationally expert and non-expert researchers.

## Background

A computational environment is a coherent interface to a set of applications that integrates them under a single paradigm. The design goal is to simplify the use and management of applications and their output. Here, we are concerned about bioinformatics applications. Many bioinformatics applications are based on scanning sequences for features relative to some database. This activity is a significant application in the field spurred by an exponentially growing sequence collection archived at national centers, now at around 65 billion bases at the National Center for Biotechnology Information (NCBI) [1]. Numerous applications are standard in these studies, for example, BLAST for sequence alignment [4], Markov models for profiling related sequences [5], and GrailEXP for searching for predicting genome features [6]. These and other programs are often integrated together in different ways, raising the challenge in bioinformatics of managing trails of data and methods applied to them. Often programs are composed together in *ad hoc *ways. This can lead to errors in interpreting results, lost time in rewriting the software *glue *to compose the programs, and overall poor solutions. The problem is not insignificant, and includes scientists from numerous disciplines involved in the interdisciplinary bioinformatics field. There are intermediate solutions to unified approaches to using bioinformatics software such as the somewhat standardized interfaces and data models provided by Bioperl and Biojava [4], but these appropriately do not attempt a user paradigm.

The BCJ is geared for bioinformatics applications where computations are often organized as workflows. A workflow consists of a series of computations connected by a flow of information between them (cf. task/control-based workflows [5]). Computational experiments are composed of workflows. As in the laboratory, computations are often repeated or modified to create new experiments to test additional hypotheses. When considering computational environments, there are several distinct factors to be considered: the execution environment, computation design, and the management of experiments and data.

A number of execution environment issues need to be considered. First, the target computing platform may be a standalone workstation, a local-area cluster, or a wide-area grid. In the wide-area grid scenario, integration of resources may be difficult because of decentralized security and administrative tasks. Second, the native execution environment may support simple, linear execution pipelines where the output of each stage drives only one input, or more complicated execution graphs where one output can branch to provide multiple inputs. Iteration and conditional branching are challenging execution features to include because there are many iteration and conditional branching strategies to choose from. Third, extension of the environment may be done with internal tools that are part of the (target) execution environment, or with external tools that a user runs on his/her local workstation separately from the execution environment. Fourth, web services can form the basis for an execution environment.

The graphical user interface paradigm for specifying the experiment design is central to the usability of the environment. Ease of use is achieved with an intuitive and consistent interface to applications, data, and results. Experiment-management issues including archiving, searching, modifying and retrieving existing workflows, searching stored data, searching annotated workflows/experiments, and the ability to export data to and import data from external sources impact the usability of the environment. Finally, other miscellaneous features, such as access controls on data, data provenance, experiment repeatability, collaboration, and annotation of experiments are important.

## Computational journal overview

The BCJ documents the entire computational experiment. The BCJ has mechanisms to record the complete context of the experiment to enable its review and re-execution. Furthermore, by recording this information, the ability to interpret results is enhanced. Although the range of computational experiments in bioinformatics research is extensive, the experimental processes have much in common. We leveraged the similarities to arrive at our framework for this domain. We chose the traditional laboratory notebook, long used by biologists to record wet lab experiments, as the base paradigm. However, the BCJ is distinct from a lab notebook. The BCJ's task is limited to providing support for managing and performing computational experiments with a complete electronic record to enable advanced functions like searching and collaboration. From the user perspective, the top-level structure of the computational journal is a collection of journal entries and relationships among these entries. More formally, an *entry *is the basic unit of information provided by the end user for the BCJ. Each entry is characterized by a single *content type *that identifies the format of this information. The number of content types is user extensible, allowing the BCJ to support new data formats and programs. Relations among entries provide an organizational structure to enable tracking entry dependencies, e.g., entry A "is an execution input" for entry B. These relationships are defined automatically as the entries are created and then they are maintained by the BCJ environment.

Our implementation of the BCJ uses the Eclipse infrastructure [7]. The Eclipse project is an open source software framework that provides a development platform for building applications. Eclipse is based on a plug-in framework that supports the requirementsdiscussed above. Eclipse provides a rigorously tested, refined, and documented infrastructure for the management of plug-ins. In addition, we were able touse several Eclipse sub-projects, such as the Graphical Editor Framework (GEF), to provide infrastructure for BCJ – specific editors. By using Eclipse, we have been able to focus directly on the development of a set of plug-ins and functionality particular to theBCJ.

**Figure 1:**
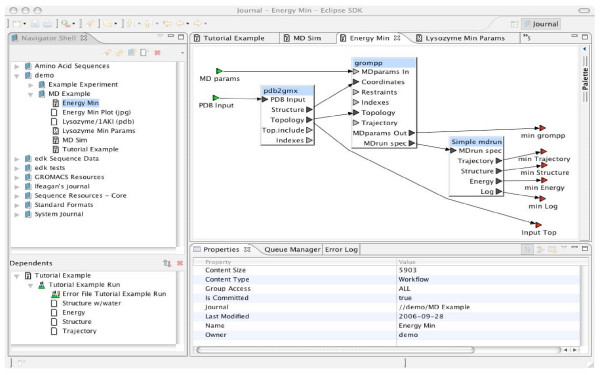
Sample BCJ Session.

The BCJ is composed of a two-tier architecture with a single server and multiple clients. The server is located on a cluster while clients run a multi-platform application supported on Windows, Linux, or OS X. The security model provides encryption between the client's computer, the BCJ database, and the cluster, as well as the concept of group-based access control. Workflows are defined graphically with the client, and the requested computations are loaded into the computational resources for execution. Tools can be added using XML and an execution wrapper script (for example, specific bioinformatics applications like BLAST). The BCJ, similar to Wildfire (see Appendix), offers a drag-and-drop interface for workflow creation (Figure 1). The BCJ does not provide iterations and conditional branches, but these are planned.

In addition to providing program integration, the BCJ manages workflows, data, and experiment definitions. Data that are input to and output from workflows are stored and linked with the corresponding execution specification, thus tracking all information related to a computational experiment. For example, users can search for experiments that used specific data as an input or created specific data as an output. Each execution of a workflow can be identified, along with the input, output, and parameters. There are also options to search based on content type, owner, journal, and user annotations of workflows and experiments to facilitate experiment creation and management. The BCJ can import data from external sources such as local files, program output, or web resources; and it can export data to a file, for other use.

The BCJ provides four of the five types of provenance described in the taxonomy of data provenance techniques developed in [8]. These four types are: (1) audit trail (complete traces of the execution can be stored); (2) replication recipes (the steps, parameters, and other transformations are recorded in experiments); (3) attribution (authentication and database control over ownership metadata ensures proper pedigree can be established); and (4) informational (annotations of varying types along with indexing of data). (The fifth type of provenance is data quality, i.e., fitness of the data for an application.) Data, workflows, and experiments can be shared through group-level access control lists. Scientists working on the same project can publish their work to other users through the BCJ. Annotation of experiments, workflow, and data can be textual or may be of other types, for instance, a Microsoft Word^® ^document or a picture of a plot related to an experiment.

### Comparisons

Four aspects were used to analyze the environments discussed above: execution environment, workflow design, data management, and additional features. A comparison based on these aspects with other computations is discussed below. (See the appendix for introductions to the systems discussed in this section: PathPort, Pegasys, PISE, BioCoRE, MIGenAS, Taverna, and Wildfire.)

#### Execution

Most surveyed solutions were designed to be run locally or on the grid–few were designed for cluster-based systems. Neither PathPort nor PISE were implemented for cluster-based execution. MIGenAS was written to be run on only one specific cluster. BioCoRE, Pegasys, Wildfire, Taverna, and BCJ allowed execution on a local cluster. Only Pegasys, Wildfire, Taverna, and the BCJ offer workflow capabilities. PISE and MIGenAS allow for linking program input and output, but neither goes beyond a serial pipeline. Furthermore, BioCoRE, MIGenAS, and Wildfire do not offer the ability to add tools to the server's execution environment, while only PathPort, Wildfire, and the BCJ allow client-side, external tools to be added. Only the BCJ and PathPort allow for both internal and external extensibility. Table 1 summarizes the execution capabilities of the environments.

**Table 1:** Execution capabilities of analyzed systems

Tool	Cluster-Based Execution	Workflow/Pipeline	Web Services	Internal Tool Extensibility	External Tool Extensibility
PathPort	No	No	Yes	Yes (Web Services)	Yes
BioCoRE	Yes	No	No	No	No
PISE	No	Pipelines	No	Yes (XML)	No
MIGenAS	Not locally	Pipelines	No	No	No
Pegasys	Yes	Workflow	No	Yes (XML)	No
Wildfire	Yes	Workflow	Yes	No	Yes
Taverna	Yes (Grid Based)	Workflow	Yes	Yes (Web Services)	No
BCJ	Yes	Workflow	No	Yes (XML)	Yes

#### Workflow Design

All of the surveyed solutions provide a GUI, however only Pegasys, Wildfire, and the BCJ provide an interactive, drag-and-drop interface. Unlike the BCJ, all surveyed solutions use input forms that are loosely coupled to the workflow on a separate page from the workflow specification. The input of parameters for each element in the BCJ is closely coupled to the associated workflow, as is discussed below. Wildfire and Taverna provide iterations and branching based on user specifications. Wildfire iterations are essentially while loops based on user-supplied imperative programs; Taverna iterations are defined graphically. Table 2 summarizes the workflow design capabilities of these environments.

**Table 2:** Workflow design of analyzed systems

Tool	Web Interface Capabilities	Drag-and-Drop Creation	Iteration/Conditional Branching
PathPort	No	No	No
BioCoRE	Yes	No	No
PISE	Yes	No	No
MIGenAS	Yes	No	No
Pegasys	No	No	No
Wildfire	Yes (Client Applet)	Yes	Yes
Taverna	No	No	Yes
BCJ	No	Yes	No

#### Data Management

PathPort, BioCoRE, and the BCJ are the only environments that provide data management. Additionally, only Pegasys, Wildfire, Taverna, and the BCJ provide workflow management. The combination of workflow and data management increases the usefulness of the environment. Table 3 summarizes the data management capabilities.

**Table 3:** Data management properties of analyzed systems

			Searching		
				
Tool	Data Management	Workflow (Experiment) Management	Data	Workflows	Data Export/Import to/from Other Tools
PathPort	VBI Curated Data	No	Yes	No	Yes
BioCoRE	Yes (BioFS)	No	No	No	Yes
PISE	No	No	No	No	Yes
MIGenAS	No	No	No	No	Yes
Pegasys	No	Yes	No	No	Yes
Wildfire	No	Yes	No	No	Yes
Taverna	No	Yes	No	No	Yes
BCJ	Yes	Yes	Yes	Yes	Yes

#### Additional Features

Of the systems reviewed, only the BCJ and Taverna provided the ability to track the provenance of data created within it and provided the ability to repeat an experiment under the same conditions. Note that for the BCJ to support experiment repeatability, program versioning needs to be implemented. This provides the ability to re-run computational experiments. The BCJ and BioCoRE encrypt information exchanges between the client and the server. Additionally, the collaborative features in several reviewed systems consisted of data sharing by exporting data/workflows to a shared file and sharing the file directly. Collaboration at the system level is offered by the BCJ. BioCoRE offered many collaborative features, including message boards and chatting, while the BCJ natively handled collaboration through the sharing of data, workflows, and experiment journals. Annotation of data and experiments, like in BioCoRE, Wildfire, Taverna, and the BCJ, is a valuable feature and is an integral part of workflow and data management. Table 4 summarizes these features.

**Table 4:** Additional features of analyzed systems

Tool	Data Provenance	Experiment Repeatability	Group Access	Encryption	Collaboration	Annotation
PathPort	No	No	No	No	Yes (Exporting Data)	No
BioCoRE	No	No	Yes	Yes	Yes	Yes
PISE	No	No	No	No	Yes (Exporting Workflows)	No
MIGenAS	No	No	No	No	No	No
Pegasys	No	No	No	No	No	No
Wildfire	No	No	No	No	Yes (Exporting Workflows)	Yes
Taverna	Yes	No	No	No	Yes (Exporting Workflows)	Yes
BCJ	Yes	Yes	Yes	Yes	Yes (Natively)	Yes

## Design and implementation of the BCJ

### Abstract data interface

There has been a proliferation of specialized tools, interfaces, and *ad hoc *scripts to handle specific, repetitive tasks in narrow domains. The generality of these tools is sometimes limited by implicit data formatting, organization, and naming conventions. The presence of these low-level details can impede a system's usefulness. In the BCJ environment, the interface has been designed to shield the user from these details; every data element is simply an entry, regardless of its specific content type, providing a uniform, high-level abstraction to the user. Users are insulated from tedious low-level abstractions such as files systems and their computational hardware resources.

The BCJ environment maintains every entry created by every user. An entry can be a bioinformatics application, a workflow, an experiment, or a data set. The underlying framework collects and maintains a set of meta-data for each entry, including its name, author, creation and modification dates, and content type. Every application that plugs into the BCJ environment is an entry and is accessible through the standard entry interface. Many BCJ functions manipulate only meta-data fields of an entry, other BCJ functions are designed to manipulate only a specific content type, and are enabled only when an entry containing this content type is selected.

The BCJ environment provides a variety of *navigators*. These are functions that provide a hierarchical view of a collection of entries. Navigators establish a virtual organization of the entries. This allows the user to see the data organized to match a specific activity, by selecting the navigator most appropriate for the task. The most basic navigator, the Journal navigator, displays entries according to the journal in which entries are created. This navigator provides a view of entries, similar to a common file hierarchy with journals corresponding to file directories. Other navigators display entries related to a specific entry. For example, the dependency navigator displays entries that depend on the selected entry.

The BCJ can be extended through the addition of navigators. As new tools and techniques are incorporated into the environment, it is likely that some will implement novel data organization schemes requiring a new navigator.

Shared access to all entries must be balanced with the individual's need for security and privacy. The BCJ security model only allows the creator of an entry to modify it. This simple model is consistent with the larger computational journal paradigm. The journal is primarily a record of experimental steps. Once completed, a step may no longer be modified, however, it may be followed by additional experimental steps to refine results and investigate variations on the base experiment. This characteristic aids in maintaining the *replication recipe *provenance. If another user wants to make changes to data or an experiment created by another user, the user may create a new entry as a copy of the original one using the SaveAs operation, thereby maintaining data pedigree.

Read access to every entry is controlled by the *group access *attribute of the entry, where a group is a set of users. Only members of the associated group are able to view an entry and its contents. All manner of access to an entry is controlled by this attribute. Search results will only contain entries for which the initiator has group access. The BCJ environment provides administrative tools to manage groups.

The BCJ environment provides another form of access control. Every entry contains a *committed *attribute. Once set, the content of the entry may no longer be modified by any user, including the entry's creator. The committed attribute of an entry can never be reset. The committed attribute is fundamental both to encourage sharing among users and to ensure reproducibility of experimental results.

### Single framework for diverse programs

A key feature of bioinformatics research is the breadth of programs that are being used by team members to address a shared goal. Frequently, specialized user interfaces are constructed around a set of closely related programs to facilitate their usage and to help perform repetitive tasks. However, integration of the process and results among these specialized interfaces is often difficult. The BCJ fundamental paradigm leverages the similar structures of computational experiments in biological experiments. These often are chained computations where the output of one is the input of another. However, the program's data formats and graphical interfaces can vary widely. Thus, within its common framework, the BCJ provides the flexibility for the problem – specific components. The BCJ environment provides common support for the *experimental process*, and *flexibility *and *extensibility *in the framework in these three key areas.

Designing a database schema broad enough to handle all forms of biological data can be a daunting undertaking. Specialized systems such as the Genomic Unified Schema (GUS) are designed for this [9]. For the BCJ role in performing and recording computational experiments, this generality is not a critical requirement. We have chosen a data model that simply stores the content of an entry as an uninterpreted stream of bytes. The BCJ domain does not attempt a structured biological database schema. Other systems exist for managing this problem with issues discussed elsewhere [9]. Instead, the BCJ uses a simple generic entry. Every entry is stored in the same table with a very simple schema. This schema consists of a fixed number of fields such as name, author, creation time, data type information, and a pointer to actual content which is stored as a vector of bytes. With this simple structure, support for a new data format simply requires the definition of a new content type.

No update or extension of a database schema is required. All BCJ environment functions that operate on the abstract entry interface, such as navigators and search tools, implicitly handle entries with content in the new format.

The BCJ supports a wide variety of raw data types. This is achieved in the storage architecture through an extensible set of *content types*. There are several parallels between requirements for Internet browsers and the BCJ environment:

1. The number of content types will grow after the product is delivered; and reissuing the product to handle new content type(s) is undesirable;

2. Each user does not require handlers for all content types;

3. There may be multiple handlers for the same content type;

4. It is desirable to distribute development of content type-specific handlers to external groups with specific interest and knowledge related to the content type.

The content types are presented and manipulated through a plug-in framework that has become ubiquitous in Internet browsers. Although a BCJ environment may contain many plug-ins, the user sees a relatively simple, coherent interface (Figure 1). The functionality that a user interface plug-in provides fits into one of four categories where each member of a category adheres to a common interface: entry navigation, entry editing, auxiliary views, and experiment execution.

From the user perspective, the BCJ provides a mechanism to navigate among the potentially large number of entries. Each navigator provides a virtual organization of the entries, based on one or more relations among the entries. This allows the user to see the data organized to match a specific activity, by selecting the navigator most appropriate for the task. At creation time, each entry is assigned a parent journal. This provides an immediate and natural hierarchical view of all entries. This journal relationship provides a view of the entries that closely resembles the file directory hierarchy familiar to all computer users. This is the most basic of the BCJ navigators. Graphically, it uses a tree-list display that is widely used on computers to present such hierarchies, but with annotations that provide more detail than a basic hierarchical file system interface.

When an entry is selected, a plug-in associated with the entry's content type is selected to edit or view the content. Graphical editors are provided for workflow definitions and experiment definitions. When a new content type is defined in the BCJ environment, it is natural to incorporate a new editor plug-in tailored for the type. Many of the program inputs and outputs in the bioinformatics domain are textual. For these types, a generic text editor plug-in can be used. The editing area in the BCJ environment is subdivided into multiple panes so that the user can conveniently compare the content of two or more entries.

Some data formats, particularly binary data formats, are supported by sophisticated and optimized viewer programs. For example, three-dimensional graphical viewers are available for molecular dynamics simulation outputs. It is not practical to convert these programs to operate directly inside the framework. The BCJ environment provides an interface that allows a user to associate an external viewer, a program installed on his/her client machine, as the default viewer for a specified content type. Whenever an entry of one of these content types is selected for viewing, the BCJ environment automatically generates a local copy of the data on the client machine and invokes the associated program with this input.

In addition to editors and navigators, the BCJ environment provides a variety of additional views that present auxiliary information to support these primary tasks, such as the Queue Manager view, which displays the progress of currently executing experiments.

Precise descriptions of BCJ programs are kept in XML documents. This description is called a *resource definition *in the BCJ environment. The Document Type Definition (DTD) for the XML data can be found in [10]. The BCJ environment currently contains about sixty programs/resource definitions (see Table 5). Many resource definitions were derived from the XML descriptions for the corresponding programs in PISE [11].

**Table 5:** Resources currently defined in BCJ

**Suite/Class**	**Resource Definitions**
Sequence search and alignment	SimpleBlastall, blastn, blastp, ClustalW, extractSequences
HMMER (biosequence analysis using hidden Markov models)	hmmAlign, hmmBuild, hmmCalibrate, hmmSearch, hmmit, hmmer2sam
GROMACS (a molecular dynamics package)	editconf, genbox, grompp, mdrun, pdb2gmx
GLIMMER (a system for finding genes in microbial DNA)	glimmer
GrailEXP (predicts exons, genes, repeats, and CpG islands)	grailAlign, grailCPG, grailExon, grailGeneAssembly, grailRepeats
EMBOSS (The European Molecular Biology Open Software Suite)	backtranseq, banana, bl2seq, btwisted, cai, chips, codcmp, coderet, compseq, cpgreport, distmat, einverted, equicktandem, fuzznuc, garnier, geecee, iep, marscan, msbar, newcoils, newcpgseek, octanol, palindrome, pepcoil, pepinfo, pepstats, primersearch, profit, prophecy, prophet, recoder, redata, shuffleseq, silent, water

### Graphical interface for experiment definition

**Figure 2:**
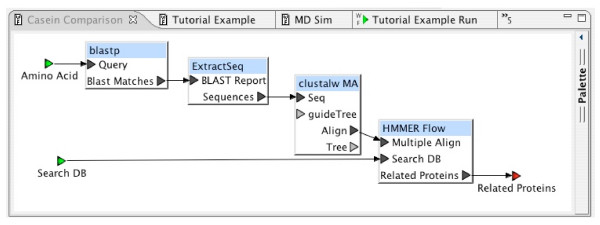
Complete Experimental Workflow.

**Figure 3:**
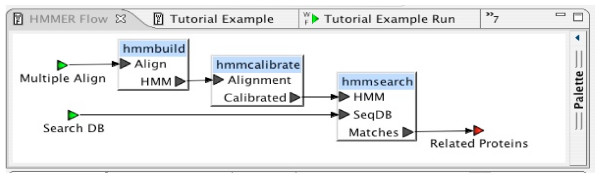
HMMER Workflow.

Experiments are defined with simple block diagrams in a hierarchical fashion. Each block represents a program, or sequence of programs, to be executed. Input and output connections are made between blocks. In the BCJ environment, a block diagram is called a *workflow*. Figure 2 is an example of a simple workflow. Workflows provide a convenient and intuitive way for any user to define a customized task. Users are not required to be familiar with programming languages to define complex tasks. Once defined, a workflow may be reused. Workflows are just another entry content type, so they can be shared among all BCJ users. Experiments can be joined by connecting resources to form workflows. A data source can be used as an input to one BCJ resource with the output directed to another resource. The BCJ also provides a mechanism to annotate workflows. Figure 3 provides an example of a simple workflow definition.

### Management of data provenance

The details of the experimental process are nearly as important as the experimental results themselves. The BCJ environment collects and maintains this information transparently, providing organizational views of data and experiments that focus on data provenance. With the provenance data maintained in the BCJ environment users can:

1. Review all the details of an experiment at anytime,

2. Regenerate the results by re-executing the experiment,

3. Run similar experiments and compare with the original results.

**Figure 4:**
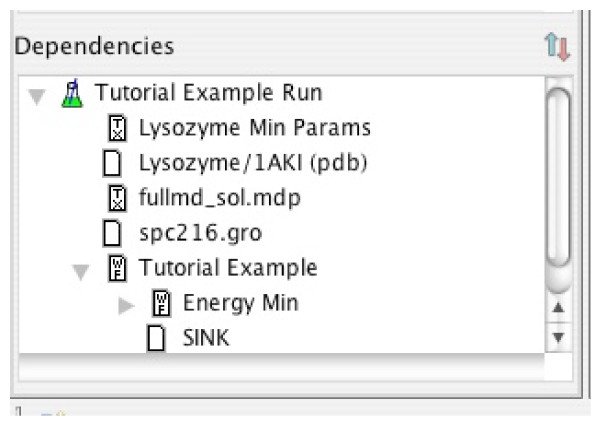
Dependency View.

When reviewing an experiment, the dependency navigator in the BCJ environment is particularly useful. The dependency navigator collects the set of entries upon which a selected entry is dependent, in a concise hierarchical view. The first level of the hierarchy contains the immediate dependencies of the selected entry. The second level of the hierarchy contains the dependencies for each of these entries, and so on. Figure 4 displays a sample dependency view.

## Computational journal experiment examples

In this section we describe two computational experiments performed in the BCJ environment, highlighting many of the features described above. The two examples were chosen from significantly different domains in order to demonstrate that the BCJ environment is sufficiently flexible to support a wide variety of applications. A user manual for the BCJ can be found in [12].

### Sequence-based investigation

To demonstrate sequence analysis in the BCJ environment, a simple example experiment was implemented that tries to identify a protein that may be a potential target for drug therapy in treating malaria. If there is sufficient divergence between some protein of the parasite and the host, then inhibition of the parasite protein is a potential target for drug therapy. In this experiment, we are interested in the divergence between the mosquito *Plasmodium falciparum *casein kinase and its vertebrate hosts. There are three applications used by the experiment: BLAST, CLUSTALW, and HMMER. The experiment involves the following computations: (1) identify similar proteins to the human casein kinase enzyme using BLAST, (2) create a global alignment using the sequences of the similar proteins that were extracted from the BLAST report using CLUSTALW, (3) construct a hidden Markov model (HMM) based on this multiple alignment, using components of the HMMER program suite, (4) run the model against the proteins in the *Plasmodium falciparum *genomic database (PlasmoDB) to identify proteins that may be functionally equivalent to human casein kinase.

In this experiment, we build two workflows. The first workflow contains the HMMER components. The second workflow, which will contain the complete experiment, will incorporate the HMMER workflow and the BLAST and CLUSTALW components.

The HMMER [9] program suite for biosequence analysis using profile HMM contains several components that are frequently used in a fixed order. In this workflow, we define a reusable component that may find value in other studies involving HMM. This workflow will construct an HMM based on the multiple alignment input, calibrate the model, and finally search a sequence database using this model.

To increase the flexibility of this computational component, the database is provided as a second input. As a design alternative, we could have chosen to specifically include PlasmoDB in this workflow (as a data source block) and have a single input to this workflow. A picture of the final workflow can be seen in Figure 3.

This workflow could be useful to a wide variety of researchers and does not contain any sensitive data or novel computation techniques, thus we set the group access control for this entry to ALL, so any user in the BCJ environment will have access to this workflow. To make the entry easier for others to find we could place the entry in a journal designated for shared workflows, and/or add an annotation briefly describing its functionality, so that other users can locate it with a search query.

In the complete workflow, we use the previously defined HMMER workflow as a *resource*. It also includes BLAST, to find proteins similar to the input protein, and CLUSTALW, to perform a multiple alignment of these similar proteins. The CLUSTALW program does not accept input in the format generated by the BLAST program, so the block, Extract Sequence, was inserted between them to perform the appropriate data conversion. Resource definitions include type information for each of their ports; the Workflow Editor does not permit connections between ports with incompatible types. These constraints help users construct only meaningful and valid workflows. Resources such as Extract Sequences serve the necessary role of automatic resolution of data reformatting. In addition, users can define new resource definitions that perform data format conversions to meet their specific requirements. The completed workflow is shown in Figure 2.

**Figure 5:**
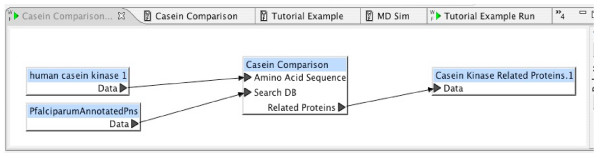
Experiment Definition.

To define an experiment based on this workflow, the user provides a protein sequence input and a database to be searched with HMMER. For this example, Amino Acid Sequence was used, for the input is human casein kinase. The Search DB input is PlasmoDB. The output file, Related Proteins, will contain the sequences from PlasmoDB that are most similar to the profile HMM built from the human casein kinase related proteins. Figure 5 shows the completed experiment definition.

When defining the experiment, the user is queried to specify access control for the experiment definition. For example, the user may want to restrict any other access to the experiment and results, by setting the access control to NONE, pending validation of results. Later, the owner can modify access control to allow access by a wider range of users. At the completion of the experiment definition, a collection of jobs to perform the tasks defined by the workflow are submitted to the computational cluster for execution. The user tracks the progress of the experiment using the Queue Manager view.

**Figure 6:**
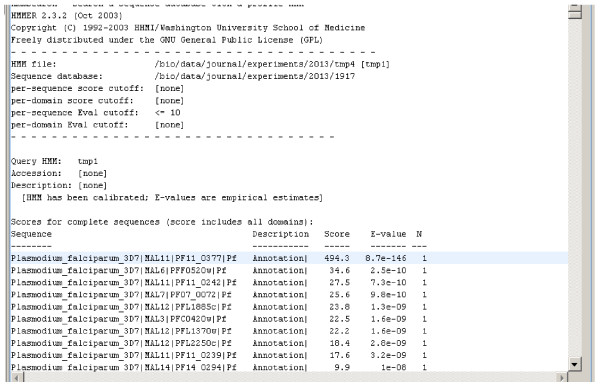
Experiment Results.

The output entry generated by the experiment contains the top sequences from PlasmoDB that match the profile created by HMMER. From the results (Figure 6), we see that the top result has the gene ID of PF11_0377. This gene happens to encode *P. falciparum's *casein kinase 1. Based on these results, we see that this kinase would not be a good candidate for drug therapy.

**Figure 7:**
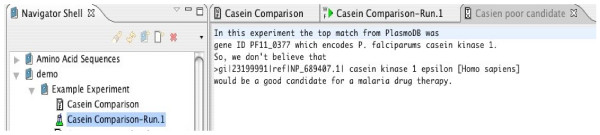
Adding an Annotation to the Experiment.

The user can summarize what has been learned and annotate the experiment as shown in Figure 7. These annotations communicate reviews to other users and provide a log for the author. Annotations also enable search capabilities, since neither the experiment definition nor the output entry directly contain the lessons learned from the experiment.

If the researcher has other candidate proteins to study, another experiment can reuse this workflow definition. Here, the user simply repeats the experiment definition step, and supplies another protein for the Amino Acid Sequence input. The BCJ environment emphasizes that providing a different sequence input is a new experiment definition, rather than a modification of the original experiment. It will be useful to keep the original experiment, even though it was not successful in locating a promising drug target. These results may be helpful months later if someone asks, "Have you considered casein kinase as a possible target for malaria?" The BCJ environment provides three alternatives to find an answer for this question:

1. The dependent viewer in the BCJ environment is particularly helpful for answering such questions. The researcher could select the casein kinase sequence entry, and find all experiments in which it is used as an input;

2. Alternatively, the researcher could use the dependency viewer, which is effectively the inverse of the dependent viewer. With this viewer, the user can select the workflow definition and immediately find all experiments, based on the workflow, that have been executed;

3. Perform a content-based search to locate an annotation that summarizes the result of a previous experiment.

### Molecular dynamics experiment

Typically, molecular dynamics (MD) simulations involve a large number of simulations, where each varies from the previous only in the input data. The organization of the computations is often tailored to some specific application such as drug design or methodology development. Analysis of the simulation results will vary widely between simulation goal and investigators' styles. In addition, although the simulations are repetitive, there are numerous places during the study where interactions with the process and output are needed. Here, we demonstrate the use of the BCJ on such a representative MD simulation. For this experiment, we use the GROMACS package [13] and an example based on a tutorial [14].

There are five major steps in this experiment: (1) converting the protein structure from a common database format, PDB (Protein Data Bank), into a format suitable for GROMACS, (2) minimizing the energy of the system, (3) solvating the protein in a simulation box with water, (4) performing an MD simulation from the initial state, and (5) analyzing the simulation results. (Often, proteins are solvated then minimized, but for purposes of demonstration, the protein is minimized first.)

Each step can be run as an independent experiment with the outputs of each step appearing as entries in the user's journal. This stepwise procedure is useful for reviewing intermediate results. Alternatively, the steps can be incorporated into a single workflow, which is useful for a tested simulation procedure. The current example represents an intermediate approach in order to demonstrate aspects of the BCJ environment; two sub-workflows are combined into another workflow to perform the end-to-end experiment.

The workflow "Energy Min" shown in Figure 1 combines the first two steps of the procedure, since these two steps are standard initialization steps. This workflow has been designed with reuse in mind. The precise parameter settings for minimization may depend on the simulated molecule (one of the inputs); thus, a second input is defined for this workflow "MD params," which will translate the simulation parameters into GROMACS format. In addition, several output ports are defined for this workflow that are not specifically required in the example here. However, these additional outputs may be of use in another application that chooses to reuse this workflow.

**Figure 8:**
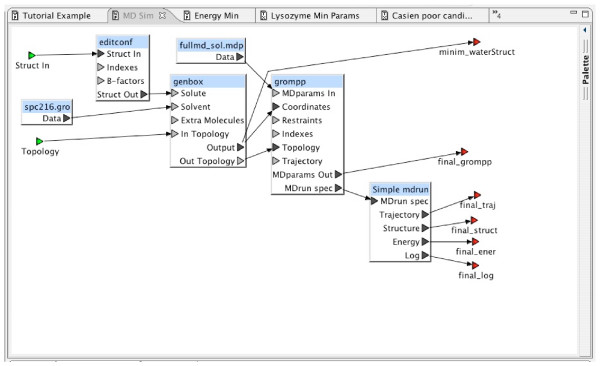
MD Simulation Workflow.

The workflow "MD Sim" shown in Figure 8 combines steps three through five of the procedure outlined above. As with the previous workflow, care was taken so that the workflow could be reused. The "Solvent" input for the resource "genbox" is provided by the data source labeled "spc216.gro." An entry for this data in the BCJ environment was created by using the "Create New Entry" interface to import this information. It contains a GROMACS-specific description of "Simple Point Charge water" that is often used as the solvent in GROMACS experiments.

**Figure 9:**
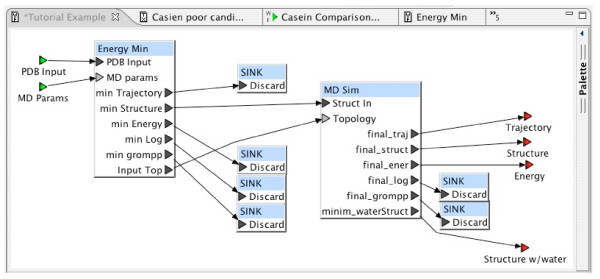
Tutorial Experiment Workflow.

The workflow "Tutorial Example" shown in Figure 9 represents the complete process from the GROMACS tutorial. It makes use of the two supporting workflows described in the previous sections, demonstrating the hierarchical workflow definition integral to the BCJ environment. As described above, the supporting workflows were not designed only for this example tutorial. Each was designed generally, so that it could be used as a component in other molecular dynamics studies. Outputs not needed can be discarded by connecting each of them to a "Sink" block. The Workflow Editor requires these explicit connections since the ports to which they are connected were required. This consistency check by the Workflow Editor helps to ensure workflow correctness.

**Figure 10:**
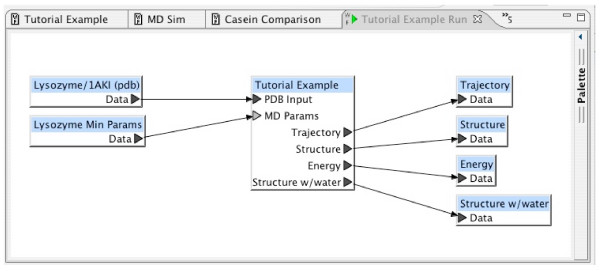
Experiment Definition.

The workflow "Tutorial Example" defines only the *process *abstracted from the GROMACS tutorial. To begin a new project (or experiment), the user must import the original input data from an external source such as a public database. When data is imported to the BCJ, an entry is created and its content is initialized to that of a file provided by the user. The BCJ physically copies the data into the environment with subsequent references to the data in the BCJ through the entry. Thus, this experiment began by having the user import a number of files into the BCJ. Consequently, the workflow, Tutorial Example, allows one to perform this experiment for any protein from the PDB. Figure 10 displays the specific experiment definition corresponding to the example in the tutorial. The protein supplied as input is lysozyme, a serine protease. The MD parameters for the energy minimization are those supplied in the tutorial.

Numerous specialized programs have been developed for molecular visualization and interaction with molecular dynamics simulation. Rather than attempt to develop another molecular visualization subsystem directly in the BCJ environment (and then require BCJ users to become familiar with a new user interface), the BCJ environment invokes an existing tool, integrated as an external viewer, to display these results. For example, VMD, Visual Molecular Dynamics [15], can be used to display the output structure.

## Conclusion

Bioinformatics research involves computational processing of multi faceted biological data at scales from atomic level to cellular. Sophisticated environments are needed to support the diverse and expanding range of computational methods, scales, models, and data. The inherently collaborative work requires managing data and methods between colleagues and public-domain databases. Complex experiments are often involved, which need to be checked, altered, and documented. The Bioinformatics Computational Journal (BCJ) reported here provides support for these activities. The BCJ handles data management and process specification in a single framework, demonstrated here with two representative bioinformatics problems. The system was founded on Eclipse, providing a rigorously tested and maintained software infrastructure. The user paradigm was designed to resemble a laboratory notebook, inline with the target users. We found this paradigm a natural basis for describing computational experiments. Features are provided in the BCJ to authenticate and establish pedigree of experiments. This is a crucial feature toward verifying and documenting computational results. Future efforts include adding iteration and branching functionality and enabling for grid collaborations.

## Competing interests

The author(s) declare that they have no competing interests.

## Authors' contributions

LF designed and developed the data management architecture for the BCJ. JR contributed to database development. AG and HA contributed the computational examples. EK was senior software architect for the BCJ. DJ contributed to the database and computing architecture. AH provided cluster computing support for the BCJ. TC and GL provided bioinformatics direction. GM conceived of the Computational Journal architecture and directed the software development team. VF was the project principal investigator, and coordinated and contributed to the development of the BCJ and the writing of this paper. All authors read and approved the final manuscript.

## Appendix of computational environments

**PathPort **[16], developed by the Virginia Bioinformatics Institute (VBI), is designed to combine information about pathogens with analysis and visualization tools. It uses web services to provide remote execution of independent, single process jobs in a non-cluster environment (it is grid-based, hence designed for execution over a heterogeneous, geographically distributed network of individual computers shared by users).

**BioCoRE **[17], developed by the Theoretical and Computational Biophysics Group at the University of Illinois at Urbana-Champlain, is designed as a biological collaborative environment. Through a combination of web pages and Java applets, BioCoRE combines the capabilities of project management, instant message, message board, lab notebook, and file sharing software with several specific applications, all designed for the simulation and visualization of molecular dynamics experiments, including NAMD [18], VMD (Visual Molecular Dynamics) [19], and MDTools [11]. BioCoRE also allows the submission of jobs to a cluster through a web page.

**PISE **[11], developed by the Pasteur Institute, is designed to offer a web interface to bioinformatics programs described in XML. The tools in PISE were not designed for execution on a cluster. (However, as part of this work some of the PISE applications have been modified to facilitate cluster computing.) The basic functionality of PISE is designed to execute programs sequentially. However, there is a plug-in called G-Pipe, which allows pipelines to be defined.

**MIGenAS **[20], the Max-Planck Integrated Gene Analysis System, is similar to PISE in that it offers a web page providing the ability to graphically enter program parameters and execute linear pipelines. Unlike PISE, which can be downloaded and run locally, MIGenAS performs all operations on servers at the Max-Planck Institute. It comes with a set of tools, and at this time does not provide a mechanism to add external or internal tools.

**Pegasys **[21], developed by the University of British Columbia's Bioinformatics Center, is designed to graphically create and execute bioinformatics workflows for high-throughput analysis. It offers clients for Linux, Windows, and Macintosh. It comes with a set of analysis programs, but allows internal tool expansion through the creation of XML tool definitions.

**Wildfire **[22], like Pegasys, is developed to create and execute bioinformatics workflows. Wildfire is available as both a standalone and a web-based workflow application that runs on a local machine, on the Grid, or on a cluster. It allows drag-and-drop creation of both simple linear pipelines and complex branching workflows. Wildfire provides some forms of iteration and conditional branching.

**Taverna **[23], a commonly used standalone workflow system, was developed to provide language and software tools for workflows as a component of the myGrid project. Taverna provides the ability to create complex workflows including iteration and conditional branching, as well as a scripting language.

**Other systems **include the commercial software Pipeline Pilot [24], Ergatis [25] (which is still in development), and Biopipe [26] (which is no longer actively supported). The SciRun software has capabilities that are conceptually relevant to this work (workflow editing, procedure replication, job control, data management, etc.), but is designed for imaging rather than molecular computational biology [27]. A commercial data mining workbench from SPSS that includes a workflow editor has also been developed[28].
